# Predicting Outcomes in Patients Undergoing Pancreatectomy Using Wearable Technology and Machine Learning: Prospective Cohort Study

**DOI:** 10.2196/23595

**Published:** 2021-03-18

**Authors:** Heidy Cos, Dingwen Li, Gregory Williams, Jeffrey Chininis, Ruixuan Dai, Jingwen Zhang, Rohit Srivastava, Lacey Raper, Dominic Sanford, William Hawkins, Chenyang Lu, Chet W Hammill

**Affiliations:** 1 Washington University in St Louis St Louis, MO United States; 2 Barnes-Jewish Hospital and the Alvin J Siteman Cancer Center St Louis, MO United States

**Keywords:** pancreatectomy, pancreatic cancer, telemonitoring, remote monitoring, machine learning, wearable technology, activity

## Abstract

**Background:**

Pancreatic cancer is the third leading cause of cancer-related deaths, and although pancreatectomy is currently the only curative treatment, it is associated with significant morbidity.

**Objective:**

The objective of this study was to evaluate the utility of wearable telemonitoring technologies to predict treatment outcomes using patient activity metrics and machine learning.

**Methods:**

In this prospective, single-center, single-cohort study, patients scheduled for pancreatectomy were provided with a wearable telemonitoring device to be worn prior to surgery. Patient clinical data were collected and all patients were evaluated using the American College of Surgeons National Surgical Quality Improvement Program surgical risk calculator (ACS-NSQIP SRC). Machine learning models were developed to predict whether patients would have a textbook outcome and compared with the ACS-NSQIP SRC using area under the receiver operating characteristic (AUROC) curves.

**Results:**

Between February 2019 and February 2020, 48 patients completed the study. Patient activity metrics were collected over an average of 27.8 days before surgery. Patients took an average of 4162.1 (SD 4052.6) steps per day and had an average heart rate of 75.6 (SD 14.8) beats per minute. Twenty-eight (58%) patients had a textbook outcome after pancreatectomy. The group of 20 (42%) patients who did not have a textbook outcome included 14 patients with severe complications and 11 patients requiring readmission. The ACS-NSQIP SRC had an AUROC curve of 0.6333 to predict failure to achieve a textbook outcome, while our model combining patient clinical characteristics and patient activity data achieved the highest performance with an AUROC curve of 0.7875.

**Conclusions:**

Machine learning models outperformed ACS-NSQIP SRC estimates in predicting textbook outcomes after pancreatectomy. The highest performance was observed when machine learning models incorporated patient clinical characteristics and activity metrics.

## Introduction

Pancreatectomy is a particularly complex operation with a 90-day mortality rate over 4% and serious morbidity rates over 20%, even in high-volume centers [1,2]. In the recently completed Alliance for Clinical Trials in Oncology (ALLIANCE) trial A021101 [3] and PREOPANC [4] multicenter clinical trials, 53% and 68% of patients, respectively, experienced at least a moderate complication from pancreatectomy. When a complication occurs after a pancreatectomy, the cost of the procedure to the health care system nearly triples from US $31,809 to US $82,576 because of prolonged hospitalization, additional treatments, and readmissions [5,6]. Complications are especially morbid in patients with pancreas cancer, a frail population with a mean age of 70 years, with up to 40% of patients being malnourished on presentation [7]. Multiple studies have shown that patients with pancreatic cancer who experience a therapeutic complication have decreased overall survival and quality of life [8].

Patients undergoing pancreatectomy have an increased risk of postoperative complications if they have poor preoperative physical health and overall performance [9,10]. To evaluate patients for surgery, physicians perform a physical examination in the office. This is subjective and can be misleading [11-13]. The patient’s condition on that day may or may not be consistent with their general health. There are simple tests such as the 6-minute walk test or the Timed Up and Go test that can be used to determine a patient’s baseline physical capacity and assess if a patient is fit for the physical demands of surgery; however, these tests have not been widely adopted [11-13]. In addition, although they are more objective than a physical examination, these tests also suffer from being a single measurement at a single time point. A more widely used surgical assessment tool is the American College of Surgeons National Surgical Quality Improvement Program surgical risk calculator (ACS-NSQIP SRC) [14-16]. It uses 20 patient-specific variables to calculate the likelihood of a patient having a complication or readmission after surgery. Although these evaluation tools are helpful, there is still a major gap in the ability to objectively measure and analyze patient health status in order to determine if the patient is fit for surgery.

Recently published data have demonstrated that telemonitoring using wearable devices with a 3-axis accelerometer and photoplethysmogram sensors can provide real-time data on patient activity metrics, which can holistically capture a patient’s physical health status [17-23]. A study utilizing this technology in cohorts of patients with gastrointestinal and advanced solid malignancy undergoing chemotherapeutic treatment demonstrated an inverse association between symptom severity and patient activity, with each increase of 1000 steps per day being associated with reduced odds for severe adverse events and increased survival [24,25]. Moreover, the application of machine learning methodologies and feature engineering techniques on patient activity data have shown that human biobehavioral rhythms, semantic features, and second-order statistical features are predictors of clinical outcomes [18-23]. Prognostic models derived using machine learning methodologies in patients who underwent pancreatectomy have also been shown to perform better than traditional methods in predicting outcomes [15,16].

For patients undergoing pancreatectomy, this technology has the potential to improve patient selection. To evaluate the relationship between longitudinal patient activity bioinformatics and their effect on surgical outcomes, our team implemented a protocol in which we provided patients with wearable telemonitoring devices before undergoing pancreatectomy at our institution and evaluated predictive outcomes. Herein, we present a prospective cohort study of patients undergoing pancreatectomy over a 12-month period.

## Methods

### Study Population

From February 2019 to February 2020, eligible patients were recruited from multidisciplinary pancreas clinics. Both men and women and members of all races and ethnic groups were eligible for this trial. The inclusion criteria for our study included patients who (1) were scheduled to undergo pancreatic resection, (2) had access to a smartphone, (3) were at least 18 years of age, and (4) were able to understand and willing to sign an institutional review board (IRB)–approved informed consent document (IRB #201810002).

### Study Design

We conducted a prospective, single-center, single-cohort trial evaluating the utility of telemonitoring devices to measure daily activity in patients undergoing pancreatectomy. The device used in this study was the Fitbit Inspire HR (Fitbit, Inc), which was selected because it provides remote data access from the device with a set frequency and enhanced granularity. It is also a waterproof, inexpensive, consumer-based device and designed to be compatible with most smartphones. At the time of consent, study patients were provided with a telemonitoring device and assisted in setting it up with their smartphone. Pancreatectomy typically took place more than two weeks after surgical consent, providing a minimum of two weeks of preoperative activity metric data. All clinical practices followed the standard of care.

### Patient Activity Assessments

Our team developed software to remotely collect activity metrics from our patient telemonitoring devices that was compliant with the Health Insurance Portability and Accountability Act. This platform collected real-time patient data with 1-minute granularity. In cases of a lost connection, the wearable device saved up to 7 days of minute-to-minute activity metrics as well as accessory data (eg, battery life at last sync and time of last sync). Our informatics system performed daily audits and ran a weekly summary routine to provide the study team with the previous week’s data, including yield. Yield was tracked using the total number of heart rate data points obtained during the day as a proxy for the percentage of the day the patient was wearing the device properly.

### Patient Clinical Assessments

Patient clinical characteristics were collected, including demographics, comorbidities, and clinical presentation. ACS-NSQIP SRC risk calculations were evaluated and documented.

### Study Outcome Measurements

All outcome measurements were prospectively collected by the study team and recorded in the patient’s secure study record. All postoperative complications were coded and graded using the Modified Accordion Grading System (MAGS) [26]. The MAGS grades complications on a scale of 1 to 6, with grade 3=severe, 4=single organ system failure, 5=multiorgan system failure, and 6=death (grades 1 and 2 complications are considered nonsevere). To ensure rigor and reproducibility, surgical complications were presented and verified at a multidisciplinary pancreas conference held every week. All postoperative complications and readmissions were collected for 30 days after hospital discharge. Complications data were then used to compute the primary outcome for our study—the textbook outcome for pancreatectomy [27]. Textbook outcome was defined as the absence of postoperative pancreatic fistulae, bile leak, postpancreatectomy hemorrhage, severe complications, readmission, and in-hospital mortality. We modified our definition of textbook outcome to allow for discharging distal pancreatectomy patients with a drain on or before day 4, the standard of care in our practice.

### Data Analysis

#### Feature Engineering

To construct machine learning models based on activity metrics data, we applied feature engineering techniques to extract three types of features: statistical, semantic, and biobehavioral rhythmic features. We extracted first- and second-order statistical features from the daily step count, heart rate, and sleep time-series data [17]. The first-order statistical features used in our analysis were mean, maximum, minimum, skewness, and kurtosis. The second-order statistical features in medical data mining were co-occurrence features for which we generated energy, entropy, correlation, inertia, and local homogeneity. We then performed detrended fluctuation analysis (DFA) on the data, which evaluates long-range correlation of noisy time-series data, and used the root-mean-square deviation from the trend, namely the fluctuation, from DFA as the feature in our analysis. [17]. The semantic features collected provided summaries of the patient’s daily activity level and sleep quality. Examples of the semantic features were time in bed, minutes to fall asleep, daily sedentary time, and daily sedentary bout count. Using the previously defined methodology, we derived and calculated biobehavioral rhythm–related features from the step count and heart rate time series [18,19]. The biobehavioral rhythmic features used in our models included stability, variability, mean of the 5 least active hours each day (L5), mean of the 10 most active hours each day (M10), amplitude (M10-L5), relative amplitude ([M10-L5]/[M10+L5]) and amplitude, phase, and midline estimating statistic of rhythm (MESOR) [20,21]. Patient clinical characteristics are potentially complementary to patient activity metrics, and we incorporated that data into the predictive models. For these categorical variables, we applied standard one-hot encoding to transfer them into features that could be used together with the features extracted from the activity metrics.

To account for variation in the study participation period (ie, time to surgery), the extracted patient activity features were unified to consistent dimensions. Biobehavioral rhythmic features were computed for the entire study participation period, and the statistical and semantic features were generated daily. In order to eliminate varying input feature dimension caused by different lengths of monitoring periods, we used mean and variance of the statistical and semantic features of a participant as the final inputs to the machine learning models.

#### Machine Learning Methods and Statistical Considerations

Multiple machine learning models were developed, trained, and evaluated for their ability to predict outcomes by discovering complex underlying patterns from multimodal time-series patient activity data collected from wearable devices and patient clinical characteristics. To avoid overfitting, we performed state-of-the-art “shallow” machine learning models, including random forest, gradient boosted trees (GBT), k-nearest neighbors (KNN), support vector machine (SVM) with linear kernel, and logistic regression (LR) with L1 penalty. A GBT model is an ensemble of weak decision trees that classifies the samples based on the predictions of those trees [22]. The algorithm iteratively fits a weak decision tree to the pseudo-residuals from the last iteration. We then employed regularization and feature selection to avoid overfitting and improve generalizability of the models. When implementing the GBT model, we explored established regularization techniques including controlling the complexity of the trees, applying shrinkage during the training process, and using stochastic gradient boosting. In general, an SVM model constructs an optimal hyperplane or a set of hyperplanes that can separate the samples of different classes by enforcing a large margin. It then makes predictions by deciding which side or region of the hyperplane the input sample should be on. In our implementation, we chose a linear kernel instead of other nonlinear kernels, such as a radial basis function (RBF) kernel, because the linear kernel is less likely to be overfitted in small data sets. LR with L1 penalty enforces the coefficients of less important features to be shrunk to zero, which works well for the case that has multiple features. For the feature selection in the training phase, we implemented a mixture of feature selection methods, using the chi-square statistic as the heuristic for categorical features and the *F* statistic from analysis of variance (ANOVA) for continuous features. When training the models, the hyperparameters were tuned using grid search. For example, for SVM the kernel choice and regularization strength were tuned, for GBT the coefficients of L1 and L2 regularization terms and the learning rate were tuned, and for LR the coefficients of elastic net regularization were tuned.

Leave-one-subject-out cross-validation (LOSO CV) was used for calculating the performance metrics, such as area under the receiver operating characteristic (AUROC), sensitivity, specificity, precision, and F1 score. LOSO CV was able to evaluate the model’s performance on unseen patients, namely the out-of-sample accuracy [23]. Model explanation techniques were explored to study the relation between input features and predicted outcomes. We used the SHapley Additive exPlanations (SHAP) technique [28], which associates each feature with an importance score—the Shapley value. SHAP is an established model-agnostic explanation approach that can be used to explore models from any kind of machine learning [29].

#### Missing Data

There were three possible causes of missing data: (1) improper wearing of the device, (2) lack of user compliance (not wearing the device), and (3) loss of connectivity for longer than 7 days. For patients with missing data, we applied a two-level imputation method to the activity metrics collected by our telemonitoring devices [17]. The data-level imputation was to fill the missing data points in heart rate time series if the daily data yield, defined as the fraction of the expected data points that were successfully collected, was equal to or above the threshold (10%). The imputed time-series data were then used to compute the features [23]. We applied KNN imputation to estimate the missing heart rate data based on recent step count and heart rate data in a sliding window (eg, 5 minutes). For those heart rate time series with a daily yield of less than 10% but greater than 0%, we used feature-level imputation to directly impute their corresponding statistical and semantic features. For the feature-level imputation, we again applied KNN imputation to the missing statistical and semantic features based on other available features from the same participant on the same day. Days with no data (daily yield of 0%) were discarded in the analysis.

#### Model Performance Evaluation

To evaluate the effectiveness of the machine learning models in predicting postoperative outcomes, defined by the modified textbook outcome, we compared them with clinical patient performance status assessment tools, including the ACS-NSQIP SRC. Utilizing the ACS-NSQIP SRC as our baseline model, we evaluated the performance and efficacy of this approach and applied machine learning models to (1) patient clinical characteristics (demographics, comorbidities, and clinical presentation), (2) features derived from remotely collected activity metrics, and (3) patient clinical characteristics + features derived from remotely collected activity metrics. The comparative evaluation of the “patient activity–only” and “clinical characteristic–only” models assessed the predictive power of activity metrics, while the performance of a combined “patient activity + clinical characteristic” model, by design, tested whether activity metrics and clinical records complement each other to yield better results.

## Results

A total of 54 patients were enrolled in the study, and 48 patients completed it. Four patients had their pancreatectomy cancelled on the day of surgery because of intraoperative evidence of advanced disease, and 2 patients electively chose to withdraw for nonmedical reasons. All patients had an independent functional status. Of the 48 patients who completed the study, 29 (60%) were females and 19 (40%) were males, with an average age of 63.2 (SD 11.6) years. Patients underwent three different types of pancreatectomy, including pancreaticoduodenectomy (n=41, 85%), distal pancreatectomy (n=6, 13%), and total pancreatectomy (n=1, 2%). The surgeries were performed open in 28 (58%) cases and minimally invasively in 20 (42%) cases. Final surgical pathology was adenocarcinoma (n=36, 75%), neuroendocrine (n=7, 15%), benign disease (n=4, 8%), and metastatic renal cell carcinoma (n=1, 2%).

In our cohort, 28 (58%) patients had a textbook outcome, with the other 20 (42%) patients not achieving a textbook outcome. Fourteen patients developed 19 severe complications (MAGS score ≥3), including delayed gastric emptying (n=3), pancreatic fistula (n=3), organ space infection (n=2), postpancreatectomy hemorrhage (n=4), nonpancreatic anastomotic leak (n=1), myocardial infarction (n=1), and other (n=5). Additionally, 11 patients required readmission to the hospital. See Table 1 for univariate analyses of demographic and comorbidity features stratified by textbook outcome in our cohort.

**Table 1:** Patient characteristics.

Characteristic		Patients with complications (n=20)	Patients with textbook outcomes (n=28)	*P* value^a^
Age (years), mean (range)		67.24 (48.14-80.52)	60.26 (31.02-84.02)	.04
**Gender, n (%)**				.12
	Male	11 (55)	8 (29)	
	Female	9 (45)	20 (71)	
**Race, n (%)**				.86
	White	19 (95)	25 (89)	
	Non-White	1 (5)	3 (11)	
**Comorbidities, n (%)**				.06
	≥5	12 (60)	8 (29)	
	<5	8 (40)	20 (71)	
**Tobacco use, n (%)**				.45
	Never smoked	11 (55)	19 (68)	
	Active smoker with >10 pack years	1 (5)	3 (11)	
	Active smoker with <10 pack years	0	1 (3.5)	
	Past history of smoking with >30 pack years	7 (35)	4 (14)	
	Past history of smoking with <30 pack years	1 (5)	1 (3.5)	
**Medications, n (%)**				.48
	≥5	7 (35)	6 (21)	
	<5	13 (65)	22 (79)	
**ASA^b^** **class, n (%)**				.07
	1	0	1 (3.6)	
	2	7 (35)	18 (64.3)	
	3	13 (65)	9 (32.1)	
BMI (kg/m^2^), mean (range)		27.99 (20.30-37.00)	29.03 (19.00-48.07)	.59
**Prior surgery, n (%)**				.02
	Yes	15 (75)	10 (36)	
	No	5 (25)	18 (64)	
**Operative approach, n (%)**				.38
	Open	14 (70)	14 (50)	
	Laparoscopic	4 (20)	9 (32)	
	Robotic	2 (10)	5 (18)	
**Operation type, n (%)**				.22
	Pancreaticoduodenectomy	18 (90)	23 (82)	
	Distal pancreatectomy	1 (5)	5 (18)	
	Total pancreatectomy	1 (5)	0 (0)	

^a^*P* values were derived from chi-square tests for categorical variables and *F* tests for continuous variables.

^b^ASA: American Society of Anesthesiologists.

Patient activity metrics were collected over an average of 25.9 days (range 6 to 153 days) before surgery. The average daily yield of all patients, defined as the fraction of expected heart rate readings per minute that were successfully collected in a day, was 82.1% (SD 23.5%). High data availability was defined as days with a yield greater than or equal to 50%. Based on this, the average number of days per patient with high data availability was 19 (range 2 to 102) and the average percentage of days with high data availability per patient was 79.8% (range 14.8% to 100%). Patients took on average of 4162.1 (SD 4052.6) steps per day, had an average heart rate of 75.6 (SD 14.8) beats per minute, and had an average sleep time series of 2 (SD 1), which was a mean DFA of their sleep stages with 50-minute windows. The average ACS-NSQIP SRC calculations for a patient developing any complication was 27.3% (SD 6.4%), developing a serious complication was 23.3% (SD 5.5%), and being readmitted was 15.1% (SD 3.4%).

Utilizing the ACS-NSQIP SRC as our baseline model, we evaluated the performance and efficacy of this approach and applied machine learning models to (1) patient clinical characteristics, which included demographics, comorbidities, and clinical presentation; (2) patient activity with features derived from remotely collected activity metrics; and (3) patient clinical characteristics + patient activity with features obtained or derived from both clinical records and activity metrics. Table 2 shows the performance comparison of these models at predicting a textbook outcome. The predictive models were trained with probabilistic outputs and then the classification thresholds were adjusted to obtain a sensitivity of 0.9 in order to ensure a high detection rate and allow an equitable comparison. Our AUROC curves were 0.6333 for the ACS-NSQIP SRC, 0.7054 for the patient clinical characteristics model, 0.7027 for the patient activity model, and 0.7875 for the patient clinical characteristics + patient activity model.

**Table 2:** Performance comparison of machine learning models trained with different data sources.

		Metrics^b^				
Parameter^a^	Model	AUROC^c^ curve	Sensitivity	Specificity	Precision	F1 score
ACS-NSQIP SRC^d^		0.6333	0.9000	0.0370	0.4091	0.5625
Patient clinical characteristics	LR^e^	0.7054	0.9000	0.2321	0.4558	0.6051
Patient activity	SVM^f^	0.7027	0.9000	0.2107	0.4491	0.5992
Patient clinical characteristics + patient activity	GBT^g^	0.7875	0.9000	0.3929	0.5143	0.6545

^a^Parameters used for the models are summarized in Multimedia Appendix 1.

^b^The metrics for the machine learning models represent the average across all leave-one-subject-out cross-validation folds.

^c^AUROC: area under the receiver operating characteristic.

^d^American College of Surgeons National Surgical Quality Improvement Program surgical risk calculator (ACS-NSQIP SRC) was used as the baseline model for complications from pancreatoduodenectomy.

^e^LR: logistic regression.

^f^SVM: support vector machine.

^g^GBT: gradient boosted trees.

In our analysis, we observed that 15 out of 20 features with the highest impact discovered by SHAP were from the best performing GBT model trained on patient clinical characteristics + patient activity (see Table 3 for feature exemplars).

Finally, to determine if the amount of missing data affected the performance of the classification model, the average number of days with high data availability (again, defined as days with a yield greater than or equal to 50%) for correctly classified patients was compared with that for incorrectly classified patients. The difference in the average number of days with high data availability between correctly classified patients and incorrectly classified patients was statistically insignificant (17 days, SD 10 days, versus 25 days, SD 25 days, respectively; *P*=0.12). This suggests that the amount of missing data did not affect the performance of the classification model.

**Table 3:** Analysis of variance test statistics on the features extracted from Fitbit Inspire HR (Fitbit, Inc) data.

Features^a^		Patients with complications, mean (SD)	Patients with textbook outcomes, mean (SD)	*F*_46_	*P* value	SHAP^b^ value
**Heart rate features**						
	Variance of local homogeneity	6744.5286 (5055.2469)	13362.2921 (7545.2961)	11.1603	.002	1.2694
	Mean of correlation	31.9993 (0.0007)	31.9996 (0.0004)	2.5324	.12	0.2338
	Mean DFA^c^ of heart rate with 40-minute window	22.7418 (5.3550)	24.8816 (5.0493)	1.9086	.17	0.2214
	Mean of energy	202.1648 (192.6207)	140.9836 (71.2032)	2.2724	.14	0.2064
	Mean of skewness	1.3182 (0.4978)	1.1065 (0.4253)	2.4006	.13	0.1787
	Cosinor amplitude	6.2318 (3.3540)	7.3569 (3.6230)	1.1464	.29	0.1507
	Variance of correlation	3.3737e–7 (8.8545e–7)	9.9500e–7 (2.0791e–7)	1.7977	.19	0.1500
	Log Cosinor amplitude	2.2616 (0.6844)	2.4344 (0.6922)	0.7041	.41	0.1119
	Mean of kurtosis	6.2530 (2.2063)	5.6795 (2.4526)	0.6640	.42	0.0558
	Variance DFA of heart rate with 30-minute window	12.1549 (7.9180)	17.6321 (11.8530)	3.1035	.08	0.0476
**Step features**						
	Variance of daily sedentary bout	0.4669 (0.2638)	0.5574 (0.3587)	0.8798	.35	0.2174
	Mean of intradaily stability	0.1100 (0.0808)	0.0689 (0.0368)	5.3752	.02	0.0930
	Relative amplitude	0.2948 (0.1653)	0.2097 (0.0878)	5.0969	.03	0.0662
	Intradaily stability with 60-minute window	0.1341 (0.1034)	0.0788 (0.0559)	5.4469	.02	0.0428
**Sleep features**						
	Mean DFA of sleep stages with 50-minute window	2.8834 (0.3767)	2.9634 (0.2589)	0.7294	.40	0.0471
**Categorical features**						
	Neutrophils	50.8000 (27.5481)	31.5393 (30.4855)	4.8323	.03	0.9024
	Prior surgery	0.7500 (0.4330)	0.3571 (0.4792)	8.1374	.007	0.3428
	Calcium	9.2450 (0.4955)	9.6071 (0.6464)	4.2378	.05	0.2932
	ASA^d^ class	2.6500 (0.4770)	2.2857 (0.5249)	5.8069	.02	0.1522
	Hyperlipidemia	0.6000 (0.4899)	0.3571 (0.4792)	2.8189	.10	0.0419

^a^Statistically significant features (*P* value <.05) are listed.

^b^SHAP: SHapley Additive exPlanations.

^c^DFA: detrended fluctuation analysis.

^d^ASA: American Society of Anesthesiologists.

## Discussion

### Principal Results

Preoperative clinical evaluation and assessment for surgical candidacy plays an essential role in postoperative outcomes. Patients who are more physically fit for surgery are less likely to experience complications. To better predict which patients will have poor outcomes, several tools have been developed and implemented over the years, including physical examination, patient demographics, laboratory values, and risk calculators; however, none of these are perfect. In this study, we used wearable telemonitoring technology in conjunction with machine learning to evaluate patient activity preoperatively and assess its ability to predict surgical outcomes.

Our models included patient clinical characteristics, patient activity, and patient clinical characteristics combined with patient activity, which we then compared with predictions from the ACS-NSQIP SRC. We found that all three of our machine learning models outperformed the baseline estimations from the ACS-NSQIP SRC. As shown in the results section, the ACS-NSQIP SRC had an AUROC curve of 0.6333 for predicting a textbook outcome after pancreatectomy, which is consistent with previous reported findings of AUROC curves in national samples [30]. Machine learning models created using the same patient clinical characteristics utilized by the ACS-NSQIP SRC outperformed the ACS-NSQIP SRC, with an AUROC curve of 0.7054 for LR. This was similar to machine learning models that utilized only patient activity data collected from telemonitoring (AUROC curve of 0.7027 for SVM). The best results were achieved with machine learning models that combined patient clinical characteristics with patient activity data (AUROC curve of 0.7875 for GBT). This confirmed our hypothesis that machine learning technology can outperform the standard ACS-NSQIP SRC in predicting textbook outcomes in patients who had a pancreatectomy. In addition, patient activity metrics significantly improved the predictive power.

Within the machine learning model, we utilized SHAP scores to identify features with the greatest impact. Specifically, within heart rate features, the “variance of local homogeneity” in heart rate was significantly correlated with higher SHAP values. This suggests that particular attention should be paid to patients’ physiological status prior to surgery. Additionally, the “mean of intradaily stability” and “relative amplitude” of steps taken [18], which pertain to the subjects’ physical mobility, were also significantly associated with higher SHAP values. The definition and derivation of these features was described by Mao et al [29]. Similar to the findings of previous studies [18,21-23], incorporating patient activity data with patient clinical data increased the performance of our machine learning models. The patient clinical data that specifically improved the models’ performance included neutrophil levels, calcium levels, and a history of prior surgery. The Rotterdam Study [31] found that patients with an elevated neutrophil count in relation to lymphocyte count (neutrophil to lymphocyte ratio) were independently associated with increased morbidity and mortality. Likewise, multiple authors have also shown age-related changes in calcium metabolism and found that variations in absorption of vitamin D, as well as a decreased intake of calcium, are commonly seen in the elderly [32]; 26 (54%) of the patients in this study were aged ≥65 years at the time of surgery.

Physical activity is a targetable and modifiable behavior that has been shown to improve outcomes of cancer patients undergoing chemoradiation [33-35]. Similarly, a meta-analysis of 15 randomized controlled trials with more than 400 patients showed that prehabilitation prior to major abdominal surgery led to a significant reduction in overall and pulmonary morbidity [33].

Based on our early results, we think that the combination of patient activity metrics collected preoperatively using wearable devices and machine learning models has the potential to reliably predict operative risks. In addition, by objectively tracking activity metrics and identifying areas of weakness, the data will provide targets for preoperative optimization and allow surgeons to more efficiently engage patients in their surgical care even before they undergo a major procedure. The ultimate goal is to decrease the likelihood of postoperative complications, which we believe will have a particularly large impact on patients with pancreatic cancer, a growing population with a high proportion of elderly and frail patients.

### Limitations

The study was limited by a small sample size, which could potentially increase the risk of overfitting. However, as discussed in the methods section, multiple precautions were taken to reduce the effect of overfitting. We also acknowledge the risk for selection bias, as we recruited patients with access to a smartphone, which has the potential to exclude elderly patients and patients from lower socioeconomic groups.

### Conclusion

Machine learning models based on preliminary data outperform standard ACS-NSQIP SRC estimates when used to predict a textbook outcome after pancreatectomy. The highest performance at this task was observed when machine learning models incorporated patient clinical characteristics and activity metrics collected with wearable telemonitoring technology. In the future, this can provide physicians with real-time actionable data that can be used to modify management of patients undergoing pancreatectomy and develop interventions to increase patient activity.

## Appendix

Multimedia Appendix 1 Parameters used for feature extraction, imputation, and models.

## Abbreviations

ACS-NSQIP SRC: American College of Surgeons National Surgical Quality Improvement Program surgical risk calculator
ANOVA: analysis of variance
AUROC: area under the receiver operating characteristic
DFA: detrended fluctuation analysis
GBT: gradient boosted trees
IRB: institutional review board
KNN: k-nearest neighbors
LR: logistic regression
LOSO CV: leave-one-subject-out cross-validation
MAGS: Modified Accordion Grading System
MESOR: midline estimating statistic of rhythm
SHAP: SHapley Additive exPlanations
SVM: support vector machine
