# Supplementary material for: Predicting Outcomes in Patients Undergoing Pancreatectomy Using Wearable Technology and Machine Learning: Prospective Cohort Study
Source: J Med Internet Res. 2021 Mar 18;23(3):e23595. doi: 10.2196/23595 (PMC8074869; doi:10.2196/23595)
Supplement: Multimedia Appendix 1 [file jmir_v23i3e23595_app1.docx]

## Appendix

Table 4. Supplemental table on parameters used for feature extraction, imputation and model training.

| **Model** | **Hyper-parameter #1** | **Hyper-parameter #2** | **Hyper-parameter #3** |
| --- | --- | --- | --- |
| **SVM** | Kernel choice: linear kernel, radial basis function kernel | Regularization strength: 0.01,0.1,0.5,1.0,2.0 |  |
| **GBT** | Learning rate: 0.001, 0.01, 0.1 | The weight of l1 regularization: 0.0, 0.01, 0.1, 1.0 | The weight of l2 regularization: 0.1, 0.01, 0.1, 1.0 |
| **LR** | The elastic-net mixing parameter: 0.0, 0.01, 0.1, 0.5, 1.0 |  |  |
| **Imputation** | Yield threshold to perform imputation: 0.1, 0.2, 0.3, 0.4, 0.5 | Number of nearest neighbors in KNN imputation: 5, 6, 7, 8, 9 |  |
| **Feature selection for model trained on PCC** | Number of selected features: 20, 30, 40, 50, all |  |  |
| **Feature selection for model trained on Patient Activity** | Number of selected features: 20, 30, 40, 50, all |  |  |
| **Feature selection for model trained on PCC + Patient Activity** | Number of selected features: 70, 80, 90, 100, 110, 120, all |  |  |
